# Rasch analysis in the development of the NutriQoL® questionnaire, a specific health-related quality of life instrument for home enteral nutrition

**DOI:** 10.1186/s41687-018-0050-9

**Published:** 2018-05-30

**Authors:** Antonio Apezetxea, Lourdes Carrillo, Felipe Casanueva, Cristina de la Cuerda, Federico Cuesta, Jose Antonio Irles, Maria Nuria Virgili, Miquel Layola, Luis Lizán

**Affiliations:** 1 Organización Sanitaria Integrada Bilbao-Basurto, Bilbao, Spain; 2 Centro de Salud Victoria de Acentejo, Santa Cruz de Tenerife, Spain; 3 Department of Medicine, Universidad de Santiago de Compostela, Complejo Hospitalario Universitario de Santiago (CHUS); CIBER de Fisiopatologia Obesidad y Nutricion (CIBERobn), Instituto Salud Carlos III, Santiago de Compostela, Spain; 4 Hospital General Universitario Gregorio Marañón, Madrid, Spain; 5 Hospital Clínico San Carlos, Madrid, Spain; 6 Hospital Universitario Nuestra Señora de Valme, Sevilla, Spain; 7 Hospital Universitario de Bellvitge, L’Hospitalet de Llobregat, Barcelona, Spain; 8 Nestlé Health Science, Barcelona, Spain; 9 Outcomes’10, Universitat Jaume 1, Parc Cientific, Tecnologic i Empresarial, Edificio Espaitec 2, Campus del Riu Sec, Avenida Sos Baynat s/n, 12071 Castellon de la Plana, Castellon Spain; 10 Departamento de Medicina, Universidad Jaime I, Campus Riu Sec, Avenida Sos Baynat s/n, 12071 Castellón de la Plana, Spain

**Keywords:** Home enteral nutrition, Health-related quality of life, Rasch analysis

## Abstract

**Background:**

Home enteral nutrition (HEN) is a therapeutic method used in patients who are unable to ingest the required amounts of nutrients but retain a functional gastrointestinal tract. The objective of this study was to compose a specific questionnaire for measuring health-related quality of life (HRQoL) in HEN patients irrespective of their underlying condition and HEN route of administration.

**Methods:**

Literature review, focus groups and semi-structured interviews were used to propose an initial version of the questionnaire which was answered by 165 participants. The responses were analyzed using the Rasch methodology. Firstly, the appropriateness of response options was assessed. Then, the differential item functioning (DIF) was evaluated. Finally, the item fit statistics, infit and outfit, were determined.

**Results:**

Rasch analysis was performed on the responses given to the 43 items included in the initial questionnaire. Four items were excluded because more than 50% of respondents answered that the situation proposed did not apply to them. Seven items that showed overlapping and disordered categories were also removed. Pairwise DIF analysis were performed in subgroups defined by underlying disease and administration route. Eleven items presented DIF and were eliminated from the questionnaire. Finally, four items were deleted after analyzing the fit statistics, three of which did not fit the Rasch model and one did not belong to either of the dimensions. The final version of NutriQoL® includes 17 items.

**Conclusions:**

NutriQoL® is a useful instrument to assess the HRQoL of HEN patients with any disease and any administration route.

## Background

Home enteral nutrition (HEN) is a therapeutic method used in patients who are unable to ingest the required amounts of nutrients but retain a functional gastrointestinal tract. The main purpose of HEN is to achieve the caloric needs for the organism, providing the proper nutrients by means of the digestive tract [1, 2]. HEN allows patients to remain in their social and family environment, reducing the probability of complications related to hospitalizations, decreasing direct costs, and improving their health-related quality of life (HRQoL) [3].

HRQoL is a dynamic variable that evaluates the subjective influence of health status, health care and health prevention activities on patients’ capability of achieving and maintaining a functional status that permits attaining vital objectives and which reflects general wellbeing. HRQoL is a multidimensional concept whose essential dimensions are: physical, psychological and cognitive and social functioning [4]. The measurement of HRQoL is useful to assess changes resulting from therapeutic interventions or the course of the disease [5].

Several studies have assessed HRQoL in patients with HEN [6–11]. The majority of publications evaluated HRQoL using generic instruments such as EQ-5D, SF-12 and SF-36 [6, 7, 9–11]. A major limitation of these tools is that in spite of providing an overall assessment of HRQoL and making it possible to compare results with other populations, they are too insensitive to establish and measure the influence of aspects directly related to HEN [4, 6]. Combining this limitation with the fact that aspects such as mental health and emotions are less affected in patients receiving HEN [12, 13] would suggest that HRQoL results in patients receiving HEN evaluated with generic questionnaires may not represent the reality. On the other hand, specific questionnaires are available only for patients with a specific disease and a specific type of HEN [8]. In particular, one tool was identified that assesses the impact of enteral feeding tubes in HRQoL in patients diagnosed with head and neck cancer, the QOL-EF [8]. This questionnaire is not applicable to other HEN administration methods or other underlying conditions requiring enteral feeding.

Rasch analysis is a widely used method for the development and reduction of questionnaires [14–16] in a wide range of disciplines, including health [17–19], education [20] and psychology [21]. It is a methodology that belongs to the item response theory (IRT) and allows obtaining a questionnaire in which each item response is taken as an outcome of the independent interaction between the respondent’s abilities and the item difficulty [22]. The method provides an alternative approach that overcomes the limitations of the classical test theory (CTT) [23, 24]. Rasch methodology also allows for analyzing differential item functioning (DIF) which identifies items that do not function equally in different groups of participants [25].

The aim of this study is to develop a specific questionnaire to assess HRQoL in patients receiving HEN regardless of the disease and the administration route.

## Methods

NutriQoL® was developed between 2011 and 2012. To carry out this development, a comprehensive literature review, an expert focus group (*n* = 7) and a series of semi-structured interviews with patients (*n* = 21) and their primary caregivers (*n* = 10) were performed to identify the main dimensions of HRQoL in patients with HEN. This allowed drawing up an initial draft of the questionnaire, which we refer to as NutriQoL® version 0. Each item was structured in two parts: ‘a’ and ‘b’. Part a’ asked about the frequency of which HEN affects particular aspects of their HRQoL. This part was written trying to reflect literally the information provided by the patients in the semi-structured interviews. Part ‘b’ referred to the importance given to situations mentioned in the part ‘a’. This second part of the items was included to consider that all aspects of life are not equally important for everyone. This is the undelaying idea of questionnaires like the SEIQoL, a method which values the HRQoL from the individual perspective [26].

A second focus group meeting with the same previously consulted experts was carried out to evaluate the items included in NutriQoL® version 0. For each item, participants rated the clarity of the wording, the frequency of occurrence in routine clinical practice, the importance of the item in the HRQoL and the relevance of the item in the therapeutic decisions. Participants scored clarity, frequency, importance and relevance on a 5-point Likert scale (1 = not clear/ frequent/important/relevant and 5 = very clear/ frequent/important/relevant). The items remained in the questionnaire if clarity, frequency, relevance and importance were high, or if clarity, relevance and importance were high even though the frequency was low. In cases of high score for frequency, relevance and importance with a low score for clarity, the wording of the items was modified to improve this feature [15, 17]. High scores were those above the 25th percentile. Based on this assessment, version 1 of the questionnaire was issued.

Finally, 19 participants (patients and caregivers) responded to questionnaire version 1 face validity and feasibility measures. They gave their opinion about the clarity of wording and comprehension, the importance of items, the general appearance of the questionnaire and the time required for completion. The wording of items that received unfavorable opinion in clarity and comprehension by at least 5% of the participants was changed. Items which receive negative opinion in importance for at least 10% of participants were removed from questionnaire. The general presentation of the questionnaire was modified if at least 10% of the participants gave an unfavorable opinion. Based on these results, we drew up version 2 of the questionnaire to be used in the Rasch analysis.

### Rasch analysis

In order to scale and reduce the questionnaire items, we performed a Rasch analysis using the Partial Credit Model because it is a proper model from the IRT family for items with polytomous responses with ordered categories [22]. For this purpose, an observational, prospective and non-controlled pilot study was carried out. One hundred and sixty-five participants (150 required by the analysis [27] and an additional 10% for possible loss of data) answered version 2 of the NutriQoL® questionnaire. Participant selection criteria included having received HEN during at least one month and having given their written consent for participation. The sample selected was non-random, intentional, consecutive, proportional according to the main epidemiology of HEN (oncologic, neurological and malabsorption) and administration route (oral, ostomy and nasogastric tube) in Spain [28].

Rasch analysis constitutes a scaling and reduction procedure with the following steps. Firstly, the appropriateness of response options is assessed. Rasch models enable ensuring that the features of the parameters of the respondents and the questionnaire items are independent, i.e. each respondent’s estimated ability does not depend on the number and type of items they have answered nor does the difficulty of the items depend on the number and type of respondents who have answered them [22, 29], thus ensuring that all items work in the same way for all respondents completing them [24]. This makes Rasch analysis different from CTT, the most widely used measurement model in health sciences in the last century, in which conversely a respondent’s observed score depends on the number and difficulty of the items in the test. Thus, the number and the particular skills of the respondents influence the final score in the questionnaire [29].

Rasch estimations are represented graphically by category probability curves [16, 19]. A requirement of Rasch is that response categories must be ordered increasingly (so the lowest category shows the worst HRQoL and the highest the best HRQoL), so the curves should be displayed in the same order. Item curves with overlapped categories suggest an excess of response options that should be reduced, while disordered curves indicate that the categorization of responses does not work as intended [22]. After the reduction of response options, items whose curves remained disordered were deleted from NutriQoL®.

The second step in the reduction procedure is the assessment of DIF. The analysis of DIF is a measurement bias and refers to differences in the probability of giving a certain response between groups [16, 18, 25, 30]. The assessment was carried out between pairs of groups defined by the underlying disease (oncologic, neurological and malabsorption/others) and the administration route of HEN (oral, nasogastric tube, and ostomy) in order to obtain a questionnaire whose measures were independent of those characteristics. Within the Rasch modeling, there are different methods for DIF analysis, one of which is Andersen’s Likelihood Ratio Test [31] that was used in this study. This DIF analysis consists of comparing Rasch analysis estimations between pairs of groups. The graphical representation of each comparison using this test identified items with DIF as the ones located outside confidence intervals. Items with DIF were deleted from the questionnaire.

The last step consisted of determining the item fit statistics: infit and outfit. These statistics are based on residuals (difference between observed and predicted responses). They indicate how well each item fits the Rasch model and let us to assess the unidimensionality of the set of items analyzed. Outfit is more sensitive to unexpected responses in items which are far from person measure, whereas Infit is more sensitive to unexpected responses in items which are close to person measure [32]. The infit and outfit mean squares were converted to an approximately normalized t-statistic by the Wilson-Hilferty method to be more conveniently represented. The interval of values that determined a good fit was [− 2, 2] [33–35]. We considered that belonged to the same dimension those items whose infit and outfit t values were inside [− 2, 2]. Items whose infit t and outfit t values were located outside the mentioned interval were analyzed separately, as part of another dimension, or removed. All calculations involved in Rasch analysis were made using the eRm package of R statistical software [36, 37].

## Results

During NutriQoL® development, the number and phrasing of the items was progressively modified according to participants’ responses and statistical analysis. Initially 52 items were included in the first version of the questionnaire (v0), which were taken from the literature review, a focus group with experts and semi-structured interviews with patients. Subsequently, during the second focus group meeting with experts, the number of questionnaire items was reduced to 46 (v1), based on item frequency, importance and clarity. After the face validity and feasibility steps, 3 items were eliminated because of problems with clarity and comprehension, so the number of items was further reduced to 43 (v2) whose wording is showed in Table 1. Items that form the final version have been indicated in italics.

**Table 1:** Items description

	Wording of part a
Item 1	*I keep my habitual mealtimes because of HEN (*e.g. *breakfast, lunch, and dinner)*
Item 2	I can adjust HEN to unexpected events (e.g. leaving the house suddenly, an unscheduled visit).
Item 3	I can feed myself with HEN and I do not have to be hospitalized for that.
Item 4	I am forced to spend most of the day sitting or lying down for food because of HEN.
Item 5	*HEN suits my preferences (*e.g. *smell, temperature, and flavor).*
Item 6	I find HEN products suit my other health problems (e.g. products without sugar because I am diabetic, without salt because I am hypertensive).
Item 7	*Since I receive HEN, I move more easily; I feel more agile.*
Item 8	*HEN damages my skin (*e.g. *dryness, irritation, infection).*
Item 9	HEN lets me eat away from home.
Item 10	Since receiving HEN, I need more help with my personal hygiene (e.g. showering, dressing).
Item 11	*HEN lets me keep doing housework (*e.g. *cooking, ironing, cleaning).*
Item 12	HEN lets me enjoy my hobbies.
Item 13	HEN hinders having sex.
Item 14	Since receiving HEN, I have problems to travel several days.
Item 15	After some time receiving HEN, I can think about going back to work.
Item 16	*HEN lets me go out with my friends.*
Item 17	Since receiving HEN, I have stopped going to family celebrations (e.g. weddings, birthdays).
Item 18	*HEN prevents me from sleeping well.*
Item 19	I have a good appetite because of HEN.
Item 20	Since receiving HEN, I fend for myself; I can do more things without help.
Item 21	*Since receiving HEN, my physical appearance is improving (*e.g. *I look healthier).*
Item 22	*I worry that my body is adapting to HEN and I will not be able to feeding as before.*
Item 23	*I miss chewing and tasting food because of HEN.*
Item 24	Since receiving HEN, I find it a sacrifice to prepare food for others.
Item 25	I need help to eat because I cannot do it alone with HEN.
Item 26	My food preparation is simpler because of HEN.
Item 27	*I have physical discomfort because of HEN (*e.g. *bloating, heartburn, dry mouth, regurgitation).*
Item 28	*Getting HEN products is simple (i. e. they are available in pharmacies and I can easily get the preparation).*
Item 29	I find it unpleasant to eat with other people because of HEN.
Item 30	HEN lets me eat with my family.
Item 31	*I limit activities with my friends to those that are not food-related because of HEN.*
Item 32	I sense that others feel bad about me because of HEN.
Item 33	*Since receiving HEN, my family is more worried about my health.*
Item 34	*Since receiving HEN, my family watches over my food.*
Item 35	Since receiving HEN, I have to explain how I feed.
Item 36	Receiving HEN causes me an added problem.
Item 37	Since receiving HEN, I feel stronger and more energetic.
Item 38	Since receiving HEN, I am more worried about my health.
Item 39	Since receiving HEN, I feel sad.
Item 40	I would change the HEN administration route (e.g. From nasogastric tube to oral administration).
Item 41	*I trust I am well-nourished because of HEN.*
Item 42	*I have gained weight because of HEN.*
Item 43	I have adapted to receiving HEN.

### Rasch results

Rasch analysis was performed with the answers of the 43 specific items from version 2 of NutriQoL® provided by 165 participants. The main characteristics of the 165 participants (141 patients and 24 caregivers that responded on behalf of patients) are presented in Table 2.

**Table 2:** Main characteristics of participants interviewed for Rasch analysis

Characteristic	Description
Sex^a^ [n(%)]	
Men	104 (64.20%)
Women	58 (35.80%)
Age [Mean (SD)]	61 (15)
Charlson index [Mean (SD)]	3.26 (2.43)
Karnofsky index [Mean (SD)]	70.41 (16.99)
Main disease [n(%)]	
Oncologic	93 (56.36%)
Neurological	31 (18.79%)
Malabsorption	41 (24.85%)
HEN duration [n(%)]	
1-3 months	59 (35.76%)
4-6 months	34 (20.61%)
7-9 months	15 (9.09%)
10-12 months	15 (9.09%)
More than 12 months	42 (25.45%)
HEN function [n(%)]	
Sole source of nutrition	105 (63.64%)
Nutritional supplement	60 (36.36%)
Administration route [n(%)]	
Oral	92 (55.76%)
Ostomy	50 (30.30%)
Nasogastric tube	17 (10.30%)
Oral and ostomy	4 (2.42%)
Oral and nasogastric tube	1 (0.61%)
Nasogastric tube and ostomy	1 (0.61%)

^a^Data unavailable for 3 participants

The percentage of answers by category response in each item was calculated (Table 3). Items with a response rate of greater than 50% to the option *Does not apply to my current situation* were eliminated as they were considered not to represent the reality of the patient. Applying this criterion, items 6 (53.21%), 13 (52.56%), 15 (67.31%) and 24 (55.13%) were removed from the questionnaire.

**Table 3:** Percentage of answers by category response in each item

Items		Never (%)	Sometimes (%)	Usually (%)	Always (%)	Does not apply to my current situation (%)	No answer (%)
1	I keep my habitual mealtimes because of HEN (e.g. breakfast, lunch, and dinner)	3.84%	6.41%	32.05%	51.92%	5.13%	0.64%
2	I can adjust HEN to unexpected events (e.g. leaving the house suddenly, an unscheduled visit).	6.41%	25.64%	17.31%	39.74%	9.61%	1.28%
3	I can feed myself with HEN and I do not have to be hospitalized for that.	3.85%	1.28%	6.41%	71.15%	15.38%	1.92%
4	I am forced to spend most of the day sitting or lying down for food because of HEN.	39.74%	10.90%	14.74%	12.82%	19.87%	1.92%
5	HEN suits my preferences (e.g. smell, temperature, and flavor).	6.41%	12.82%	23.72%	31.41%	25.00%	0.64%
6	I find HEN products suit my other health problems (e.g. products without sugar because I am diabetic, without salt because I am hypertensive).	9.62%	1.92%	8.33%	24.36%	*53.21%*	2.56%
7	Since I receive HEN, I move more easily; I feel more agile.	10.26%	16.03%	21.79%	28.85%	21.15%	1.92%
8	HEN damages my skin (e.g. dryness, irritation, infection).	52.56%	12.82%	1.92%	1.28%	30.13%	1.28%
9	HEN lets me eat away from home.	17.95%	18.59%	7.69%	39.10%	14.74%	1.92%
10	Since receiving HEN, I need more help with my personal hygiene (e.g. showering, dressing).	53.85%	6.41%	5.13%	6.41%	27.56%	0.64%
11	HEN lets me keep doing housework (e.g. cooking, ironing, cleaning).	5.13%	14.10%	10.90%	31.41%	37.18%	1.28%
12	HEN lets me enjoy my hobbies.	5.77%	18.59%	16.03%	38.46%	19.23%	1.92%
13	HEN hinders having sex.	32.05%	4.49%	2.56%	5.77%	*52.56%*	2.56%
14	Since receiving HEN, I have problems to travel several days.	42.95%	8.33%	3.21%	15.38%	28.85%	1.28%
15	After some time receiving HEN, I can think about going back to work.	13.46%	8.33%	2.56%	5.77%	*67.31%*	2.56%
16	HEN lets me go out with my friends.	10.26%	13.46%	14.10%	44.23%	16.67%	1.28%
17	Since receiving HEN, I have stopped going to family celebrations (e.g. weddings, birthdays).	50.00%	12.82%	5.77%	12.82%	16.67%	1.92%
18	HEN prevents me from sleeping well.	69.23%	12.18%	0.64%	1.92%	14.74%	1.28%
19	I have a good appetite because of HEN.	16.67%	28.85%	19.23%	23.72%	10.90%	0.64%
20	Since receiving HEN, I fend for myself; I can do more things without help.	9.62%	13.46%	25.00%	24.36%	26.92%	0.64%
21	Since receiving HEN, my physical appearance is improving (e.g. I look healthier).	6.41%	19.87%	25.00%	42.95%	5.13%	0.64%
22	I worry that my body is adapting to HEN and I will not be able to feed as before.	21.15%	17.31%	7.05%	22.44%	30.77%	1.28%
23	I miss chewing and tasting food because of HEN.	14.74%	12.82%	5.77%	32.05%	33.33%	1.28%
24	Since receiving HEN, I find it a sacrifice to prepare food for others.	26.28%	8.33%	3.85%	4.49%	*55.13%*	1.92%
25	I need help to eat because I cannot do it alone with HEN.	47.44%	4.49%	3.85%	10.90%	32.05%	1.28%
26	My food preparation is simpler because of HEN.	14.10%	10.90%	5.77%	26.28%	39.74%	3.20%
27	I have physical discomfort because of HEN (e.g. bloating, heartburn, dry mouth, regurgitation).	46.15%	35.26%	5.77%	7.05%	5.13%	0.64%
28	Getting HEN products is simple (i. e. they are available in pharmacies and I can easily get the preparation).	5.77%	5.77%	15.38%	67.31%	4.49%	1.28%
29	I find it unpleasant to eat with other people because of HEN.	53.85%	9.62%	3.85%	8.97%	21.79%	1.92%
30	HEN lets me eat with my family.	14.74%	13.46%	8.97%	46.15%	14.10%	2.56%
31	I limit activities with my friends to those that are not food-related because of HEN.	38.46%	10.90%	8.97%	14.10%	25.00%	2.56%
32	I sense that others feel bad about me because of HEN.	50.64%	14.74%	7.05%	7.05%	18.59%	1.92%
33	Since receiving HEN, my family is more worried about my health.	44.23%	17.31%	10.26%	19.23%	7.69%	1.28%
34	Since receiving HEN, my family watches over my food.	14.10%	19.23%	14.10%	44.23%	7.69%	0.64%
35	Since receiving HEN, I have to explain how I feed.	37.82%	31.41%	8.33%	5.13%	15.38%	1.92%
36	Receiving HEN causes me an added problem.	60.90%	17.31%	7.05%	6.41%	7.05%	1.28%
37	Since receiving HEN, I feel stronger and more energetic.	7.05%	23.72%	23.72%	39.10%	5.13%	1.28%
38	Since receiving HEN, I am more worried about my health.	43.59%	22.44%	8.97%	11.54%	10.90%	2.56%
39	Since receiving HEN, I feel sad.	60.26%	21.15%	3.85%	3.85%	8.97%	1.92%
40	I would change the HEN administration route (e.g. From nasogastric tube to oral administration).	46.79%	5.13%	0.64%	9.62%	34.62%	3.20%
41	I trust I am well-nourished because of HEN.	1.28%	2.56%	12.82%	80.14%	1.92%	1.28%
42	I have gained weight because of HEN.	11.54%	28.21%	17.31%	36.54%	3.21%	3.20%
43	I have adapted to receiving HEN.	2.56%	6.41%	23.72%	64.10%	1.28%	1.92%

One of the requirements of Rasch methodology is that item response categories must be ordered in increasing level of HRQoL. To accomplish this criterion where a higher score refers to higher HRQoL, category responses of the 43 items were codified as follows:

- Items 1, 2, 3, 5, 7, 9, 11, 12, 16, 19, 20, 21, 26, 28, 30, 37, 41, 42 and 43: Never = 0, Sometimes = 1, Usually = 2 and Always = 3.
- Items 4, 8, 10, 14, 17, 18, 22, 23, 25, 27, 29, 31, 32, 33, 34, 35, 36, 38, 39 and 40: Never = 3, Sometimes = 2, Usually = 1 and Always = 0.

**Fig. 1:**
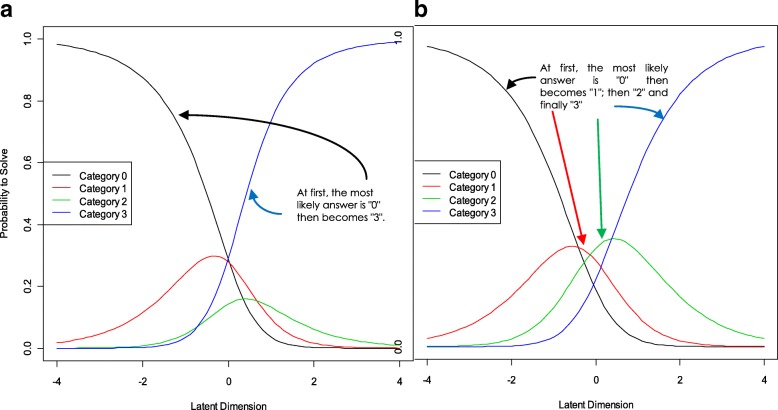
Category probability curves for items 4 (**a**) and 5 (**b**)

Category probability curves were represented for the remaining 39 items (43 minus 4) to assess the appropriateness of response options. Figure 1 shows the estimates of the thresholds corresponding to items 4 (Fig. 1a) and 5 (Fig. 1b). Curves of item 4 show overlapping, however in item 5, category curves are ordered correctly without overlapping, thus fulfilling the requirements of Rasch analysis. As most of items showed overlapping, we decided to reduce the number of category responses, so options *Sometimes* and *Usually* were unified in one category. The new categorization was:

- Items 1, 2, 3, 5, 7, 9, 11, 12, 16, 19, 20, 21, 26, 28, 30, 37, 41, 42 and 43: Never = 0, Sometimes = Usually =1, and Always = 2.
- Items 4, 8, 10, 14, 17, 18, 22, 23, 25, 27, 29, 31, 32, 33, 34, 35, 36, 38, 39 and 40: Never = 2, Sometimes = Usually 1, and Always = 0.

Another Rasch analysis was performed to assess the new categorization of items and their results showed that 32 of the 39 items presented the curves ordered correctly. The 7 items that showed overlapping and disordered categories were removed from NutriQoL®.

**Fig. 2:**
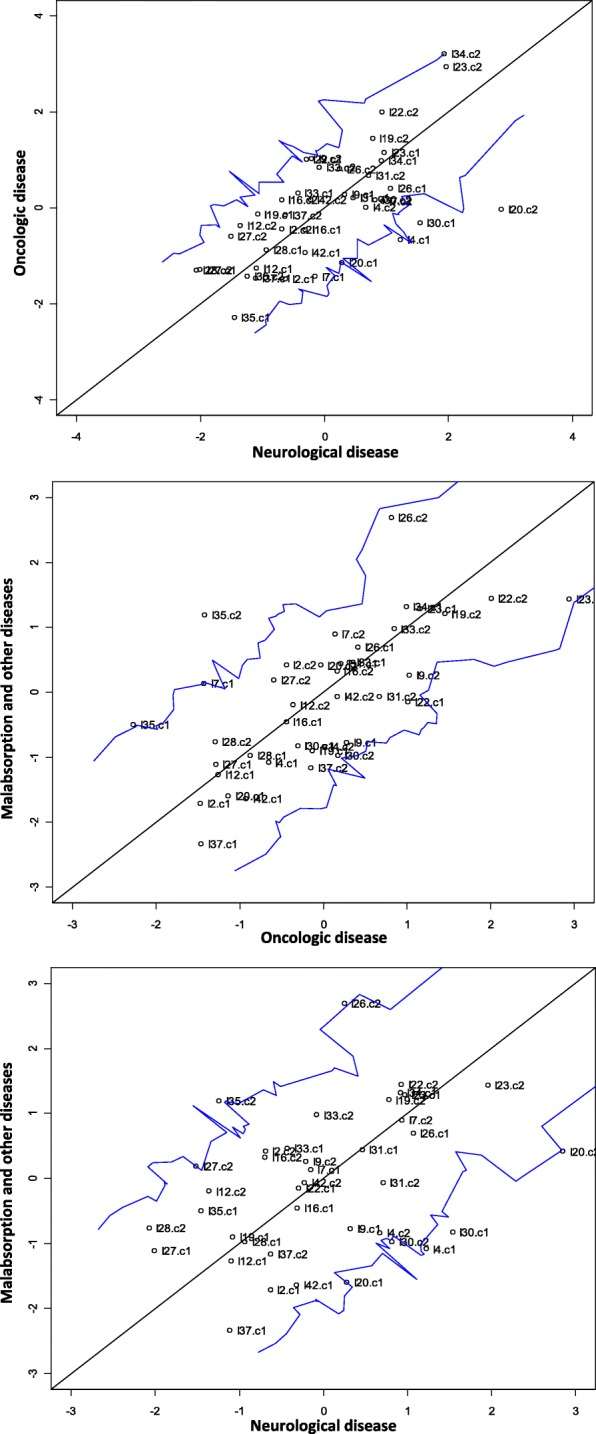
Differential item functioning in groups defined by the underlying disease (oncologic vs. neurological, malabsorption vs. oncologic and malabsorption vs. neurological disease)

**Fig. 3:**
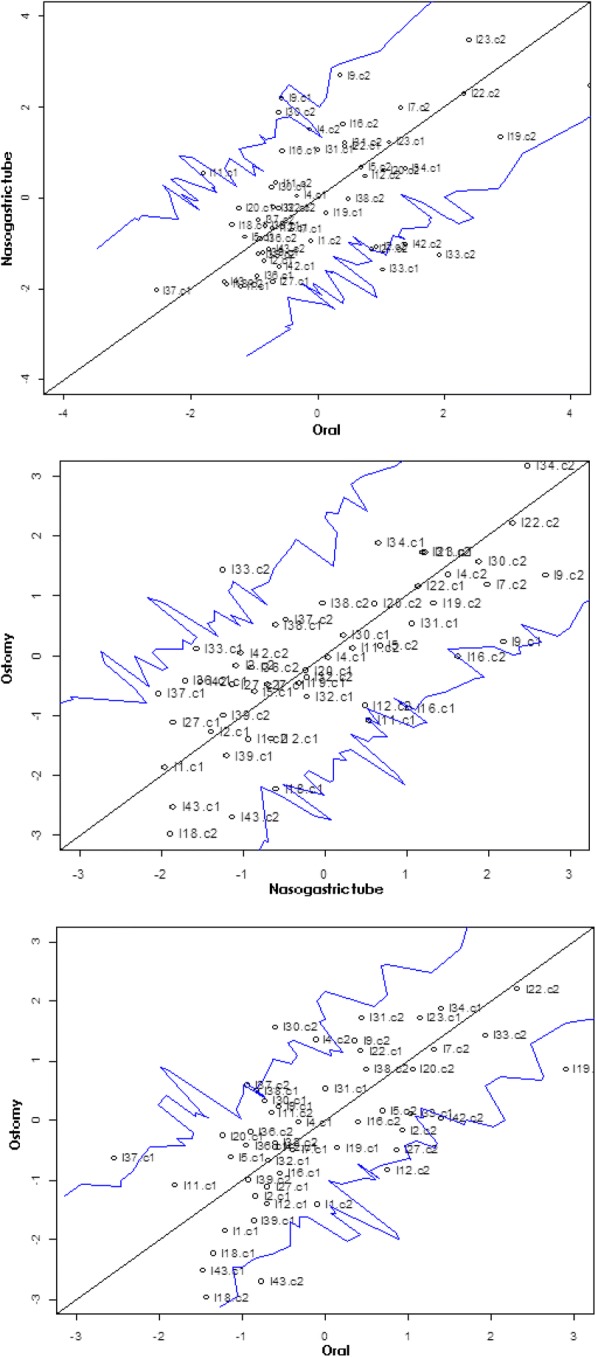
Differential item functioning in groups defined by administration route (nasogastric tube vs. oral, ostomy vs. nasogastric tube and ostomy vs. oral)

Analysis of DIF was performed with the remaining items (32 items). Figures 2 and 3 show the graphical representation of pair comparisons of groups defined by underlying disease and administration route, respectively. Eleven items (4, 9, 12, 14, 19, 20, 26, 30, 35, 37 and 43) were located outside the confidence intervals (blue lines), i.e. the ones with DIF, and were eliminated from the questionnaire.

The last stage involved in Rasch analysis was the assessment of item fit statistics (infit t and outfit t). A new Rasch analysis was performed with the 21 remaining items. As a result, 14 items (1, 5, 7, 8, 11, 18, 21, 22, 23, 27, 28, 34, 31, 41 and 42) showed values of infit t and outfit t within the range [− 2, 2]. Dimension 1 of the questionnaire was constituted by this group of 14 items. Another Rasch model was applied with the seven items that showed misfit in the previous analysis. The goodness of fit of the new analysis presented acceptable values of infit t and outfit t statistics in three items (16, 31 and 38) which constituted dimension 2. A new analysis showed that the four remaining items did not fit the Rasch model with the exception of item 2, though it did not belong to any dimension (Table 4). These four items were deleted from NutriQoL®.

**Table 4:** Infit and outfit statistics

Items		Dimension	Infit t	Outfit t
1	*I keep my habitual mealtimes because of HEN (*e.g. *breakfast, lunch, and dinner)*	1	−1.04	−1.03
5	*HEN suits my preferences (*e.g. *smell, temperature, and flavor).*	1	0.02	0.61
7	*Since I receive HEN, I move more easily; I feel more agile.*	1	−1.84	−1.77
8	*HEN damages my skin (*e.g. *dryness, irritation, infection).*	1	1.19	0.49
11	*HEN lets me keep doing housework (*e.g. *cooking, ironing, cleaning).*	1	−1.57	−1.67
18	*HEN prevents me from sleeping well.*	1	0.15	−0.93
21	*Since receiving HEN, my physical appearance is improving (*e.g. *I look healthier).*	1	−1.84	−1.68
22	*I worry that my body is adapting to HEN and I will not be able to feed as before.*	1	0.30	0.49
23	*I miss chewing and tasting food because of HEN.*	1	0.65	0.79
27	*I have physical discomfort because of HEN (*e.g. *bloating, heartburn, dry mouth, regurgitation).*	1	−1.95	−1.45
28	*Getting HEN products is simple (i. e. they are available in pharmacies and I can easily get the preparation).*	1	1.25	1.42
34	*Since receiving HEN, my family watches over my food.*	1	−1.38	−1.15
41	*I trust I am well-nourished because of HEN.*	1	0.51	0.61
42	*I have gained weight because of HEN.*	1	−1.13	−1.40
16	*HEN lets me go out with my friends.*	2	−1.24	−1.36
31	*I limit activities with my friends to those that are not food-related because of HEN.*	2	−1.32	−1.43
33	*Since receiving HEN, my family is more worried about my health.*	2	−1.05	−1.32
2	I can adjust HEN to unexpected events (e.g. leaving the house suddenly, an unscheduled visit).	Without dimension	1.86	1.81
32	I sense that others feel bad about me because of HEN.	Without dimension	−3.57	−4.59
36	Receiving HEN causes me an added problem.	Without dimension	−3.64	−4.37
39	Since receiving HEN, I feel sad.	Without dimension	−3.00	−2.92

As a result of the reduction procedure by means of Rasch analysis, the final version of NutriQoL® was composed of 17 pairs of items divided into two dimensions whose name was established according to the content of the items that were part of each one: 1) physical functioning and activities of daily living, and 2) social life aspects. These are usual dimensions of HRQoL in HEN patients [9].

**Fig. 4:**
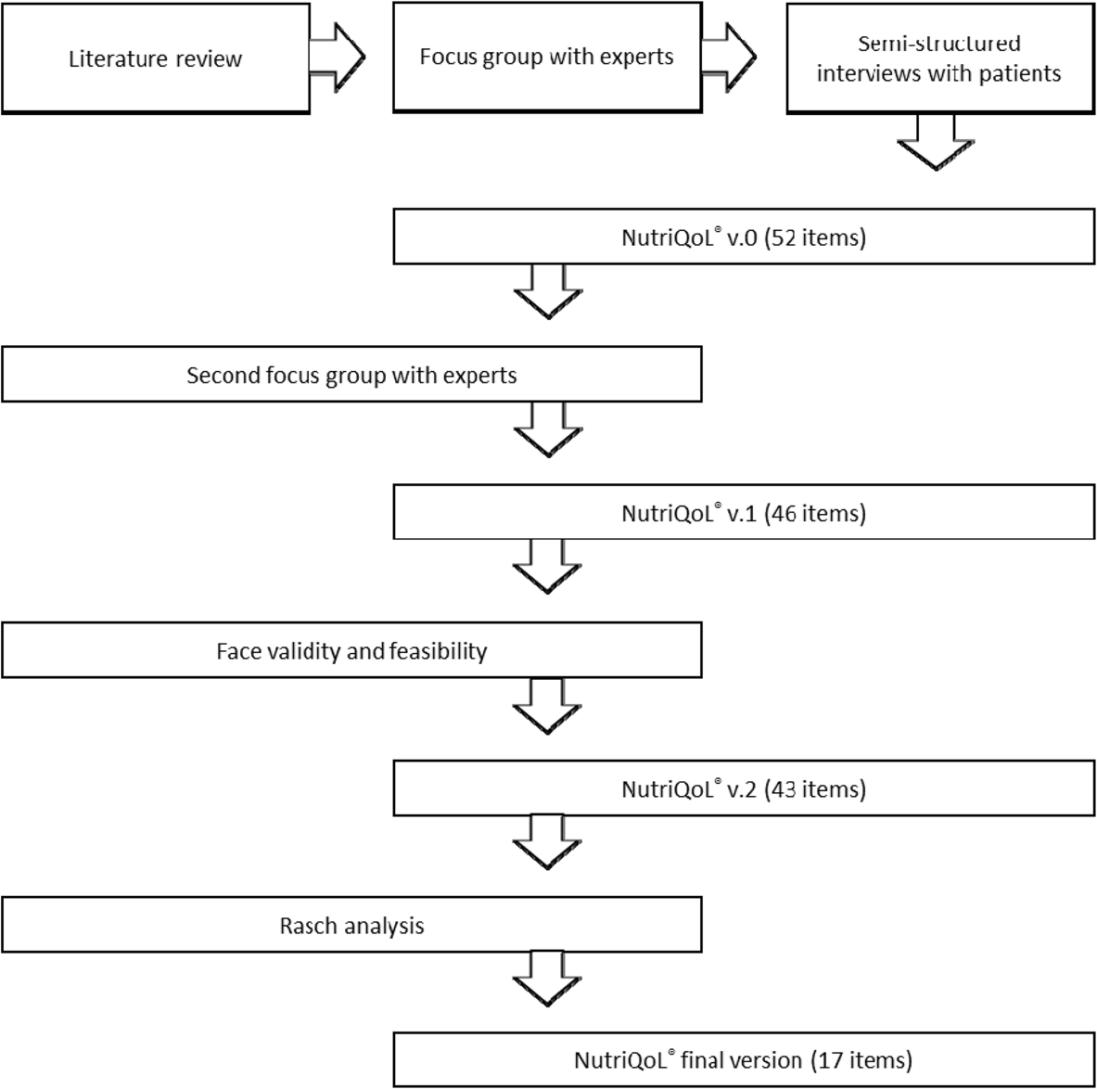
Development of the NutriQoL questionnaire

The different stages of the questionnaire development and item reduction are detailed in Fig. 4.

## Discussion

In this study, the investigators present the use of Rasch analysis to develop NutriQoL®, a novel questionnaire to measure HRQoL in patients with HEN, irrespective of their underlying condition and route of administration. Throughout a literature review, only one specific tool, the QOL-EF questionnaire, was identified to assess the impact of enteral feeding tubes in HRQoL. However, the QOL-EF is specific for patients with head and neck cancer [8].

Previous studies have assessed the HRQoL on patients receiving HEN by means of the widely used generic questionnaires SF-12, EQ-5D or the SF-36 [6, 7, 9–11]. The SF-12 questionnaire was used to measure the HRQoL in patients with percutaneous endoscopic gastrostomy [7]. The results showed that the questionnaire scores did not reflect some of the patients’ problems, such as the nausea they constantly experienced. SF-12 was also used in a study that aimed to assess HRQoL in patients with home enteral tube feeding [11]. Authors measured patients’ HRQoL at two and ten weeks after discharge, however, the results did not show differences between the two time points. Furthermore, the results and findings of the study could not be generalizable due to the small sample size. Another study used the questionnaires SF-36 and EQ-5D to evaluate the HRQoL in patients using long term HEN [9]. Subgroup analyses were performed based on the age and cancer diagnosis of patients. Results from EQ-5D did not reflect differences between subgroups, while results from SF-36 only showed statistical differences in physical functioning and role-emotional in age and cancer subgroups, respectively. Authors attributed the lower sensitivity of EQ-5D to the smaller number of items and the small sample size, in fact, they stated that their results did not represent the HRQoL of HEN patients. The questionnaire EQ-5D was also used in another study that assessed the HRQoL in patients with HEN [10]. Authors highlighted the lack of specific and relevant validated measurement tools to evaluate the HRQoL in this type of patients. They stated that the measures of HRQoL obtained by means of specific tools would be necessary to detect specific aspects of illnesses or treatments. These findings highlight the need for an instrument like NutriQoL to measure HRQOL in patients receiving HEN irrespective of their underlying condition and route of administration.

The main limitation of the study is the sample size. Although the sample size is appropriate for Rasch analysis [27], when the sample was divided into subgroups to perform the DIF analysis, the size of them were very small and DIF have limitations in this kind of situations. This weakness will be the focus of future research in order to improve the NutriQoL® results and assess the properties of the items that are part of it.

Additional analyses were carried out to ensure the reproducibility of the results (reliability), NutriQoL®^‘^s sensitivity to changes in patients’ health (responsiveness) and the reliability of answers between patient and caregiver (inter-observer reliability). These analyses were performed in a prospective study between 2013 and 2014 whose results are detailed in other publication [38]. Therefore, NutriQoL®, it is a useful instrument to assess the HRQoL of HEN patients with any disease and any administration route in a context were such an instrument was not previously available.

## Conclusion

A new tool has been developed to assess the impact of HEN in patients’ HRQoL (the NutriQoL® questionnaire). Rasch methodology has allowed performing a short questionnaire composed of 17 items able to measure HEN-related HRQoL irrespective of the patients’ underlying disease and the route of administration. The NutriQoL® questionnaire provides a specific instrument that may be used in clinical practice to adjust treatments according to HRQoL results.
